# Identification of a variant in *NDP* associated with X-linked retinal dysplasia in the English cocker spaniel dog

**DOI:** 10.1371/journal.pone.0251071

**Published:** 2021-05-04

**Authors:** Hannah Joyce, Louise M. Burmeister, Hattie Wright, Lorraine Fleming, James A. C. Oliver, Cathryn Mellersh

**Affiliations:** 1 Department of Ophthalmology, Dick White Referrals, Six Mile Bottom, Cambridgeshire, United Kingdom; 2 Department of Canine Genetics, Kennel Club Genetics Centre, Animal Health Trust, Kentford, Newmarket, United Kingdom; 3 Department of Ophthalmology, Centre for Small Animal Studies, Animal Health Trust, Kentford, Newmarket, United Kingdom; University of Florida, UNITED STATES

## Abstract

**Purpose:**

Three related male English Cocker Spaniels (ECS) were reported to be congenitally blind. Examination of one of these revealed complete retinal detachment. A presumptive diagnosis of retinal dysplasia (RD) was provided and pedigree analysis was suggestive of an X-linked mode of inheritance. We sought to investigate the genetic basis of RD in this family of ECS.

**Methods:**

Following whole genome sequencing (WGS) of the one remaining male RD-affected ECS, two distinct investigative approaches were employed: a candidate gene approach and a whole genome approach. In the candidate gene approach, *COL9A2*, *COL9A3*, *NHEJ1*, *RS1* and *NDP* genes were investigated based on their known associations with RD and retinal detachment in dogs and humans. In the whole genome approach, affected WGS was compared with 814 unaffected canids to identify candidate variants, which were filtered based on appropriate segregation and predicted pathogenic effects followed by subsequent investigation of gene function. Candidate variants were tested for appropriate segregation in the ECS family and association with disease was assessed using samples from a total of 180 ECS.

**Results:**

The same variant in *NDP* (c.653_654insC, p.Met114Hisfs*16) that was predicted to result in 15 aberrant amino acids before a premature stop in norrin protein, was identified independently by both approaches and was shown to segregate appropriately within the ECS family. Association of this variant with X-linked RD was significant (P = 0.0056).

**Conclusions:**

For the first time, we report a variant associated with canine X-linked RD. *NDP* variants are already known to cause X-linked RD, along with other abnormalities, in human Norrie disease. Thus, the dog may serve as a useful large animal model for research.

## Introduction

Retinal dysplasia (RD) is a developmental disorder that is defined as abnormal differentiation of the retina with proliferation of one or more of its components [1]. It has been described in multiple dog breeds and can be subdivided into focal, multifocal, geographic and total retinal dysplasia types [2]. RD can present as a nonsyndromic form such as that described in the Bedlington and Sealyham Terriers or it can present as a syndromic RD such as in oculoskeletal dysplasia described in the Labrador Retriever, Samoyed and Northern Inuit dog [3–7]. Genetic variants associated with oculoskeletal dysplasia have been identified in all of these breeds [7, 8]. Total RD presents clinically as total retinal detachment. This can be associated with non-attachment due to failure of contact between the neurosensory retina and retinal pigment epithelium during embryogenesis or due to complete detachment of the retina. It may also be seen in conjunction with microphthalmos, vitreal dysplasia, leukocoria and a rotary-searching nystagmus [2].

Retinal detachment has also been reported as a clinical feature in Collie Eye Anomaly (CEA) in multiple breeds [9]. CEA is characterized by regional hypoplasia of the choroid. In severe cases and especially those with colobomas, affected dogs can develop retinal detachments, intraocular haemorrhage and subsequent blindness [9, 10]. The genetic variant for CEA has been identified in non-homologous end joining factor 1 (*NHEJ1*) [11]. The mode of inheritance of total RD and CEA has been reported to be autosomal recessive in all breeds [3, 4, 7, 8, 10]. Although other retinal pathologies have been found to be X-linked in dogs, such as *XLPRA* in the Siberian Husky as well as other breeds, to the author’s knowledge an X-linked RD has not been reported before [12].

Many genetic disorders that affect humans have an equivalent disease that is recognised in the dog [13]. Retinal diseases that can result in blindness in people that are known to be X-linked include retinoschisis and *NDP* (norrin cystine knot growth factor)-related retinopathies [14, 15]. Retinoschisis can present in infants as young as two months of age but more commonly presents in males at five years and upwards with macular dysfunction. In more severe forms of the disease retinal detachment can occur with a variable reported incidence of 16.6–30% [14, 16, 17]. Variants in *XLRS1* (X-linked (juvenile) retinoschisis 1) have previously been associated with retinoschisis [18–20].

*NDP*-related retinopathies are characterised by a spectrum of fibrous and vascular changes of the retina at birth that progress and cause varying degrees of visual impairment in males. The most severe phenotype described is Norrie disease that presents clinically with a grey-yellow fibrovascular mass (pseudoglioma) secondary to retinal vascular dysgenesis and retinal detachment with congenital blindness [21]. Cognitive impairment and behavioural disturbances occur in approximately 33% and 45% of people with the disease respectively. Progressive sensorineural hearing loss usually develops, with one study revealing 85–90% of patients experiencing the onset of hearing loss by their mid-20s and cryptorchidism has also been rarely reported in cases of Norrie disease [22–24]. Associated genetic variants have been identified within *NDP*, which encodes a small, presumably secreted protein (norrin), with a cysteine-knot motif [25]. Greater than 100 different disease-causing *NDP* variants have been identified in humans to date [26]. In mice, norrin has been shown to play important roles in angiogenesis, not only in development of the eye but also the ear, brain, and female reproductive system [27].

Here we describe a novel form of RD associated with a variant in *NDP* in the English Cocker Spaniel (ECS), a small dog of the gundog breed, first recognised as a separate variety in 1893 [28]. In a family of ECS where three of the males were reported to be congenitally blind and pedigree analysis was suggestive of an X-linked mode of inheritance, we sought to investigate the genetic basis of the RD. Using two distinctive investigative approaches; a candidate gene approach and a whole genome sequencing (WGS) approach, we identified a provocative candidate variant. To the best of our knowledge, this is the first description of canine X-linked RD and the first time a variant in *NDP* has been associated with any disease in the dog. These findings also reveal the dog as a potential large animal model for studying *NDP*-related retinopathies in humans.

## Materials and methods

### Ethics statement

All dogs used for this study were privately owned pet dogs that were examined at the owners’ request following informed and written owner consent. Buccal mucosal swab sampling was performed following written owner consent and no in vivo experiments were undertaken. All clinical examinations were conducted as part of routine ophthalmic examination and not specifically for research purposes. All sample collection was approved by the Animal Health Trust Ethics Committee (04–2018).

### Animals used

In this study a total of 1260 samples of 178 breeds were used. This consisted of DNA samples from one affected ECS, nine related ECS, 170 unaffected ECS, 264 unaffected dogs, WGS data from an additional 814 canids and clinical information only from two more affected ECS. Affected dogs were defined as those with RD in the form of retinal detachment. Twelve related ECS from two litters (three affected and nine unaffected) were included in the study. DNA was collected from ten of the twelve dogs, including one affected. The remaining two affected were deceased and were therefore unavailable for DNA collection.

DNA samples from an additional 170 unaffected ECS and from 264 unaffected dogs of 93 other breeds that were previously collected for unrelated research projects were used in the candidate variant genotyping.

WGS data from 814 canids were used as ‘controls’ in the whole genome approach. These comprised two distinct groups: WGS data from 198 unaffected dogs (comprising of 98 breeds and multiple crossbreeds) previously sequenced by the authors for other studies, and WGS from 616 canids of unknown phenotypes (comprising 122 dog breeds, multiple crossbreeds, and eight wolves, of unknown clinical status) derived from the Dog Biomedical Variant Database Consortium (DBVDC) [29].

### Clinical investigation

**Fig 1:**
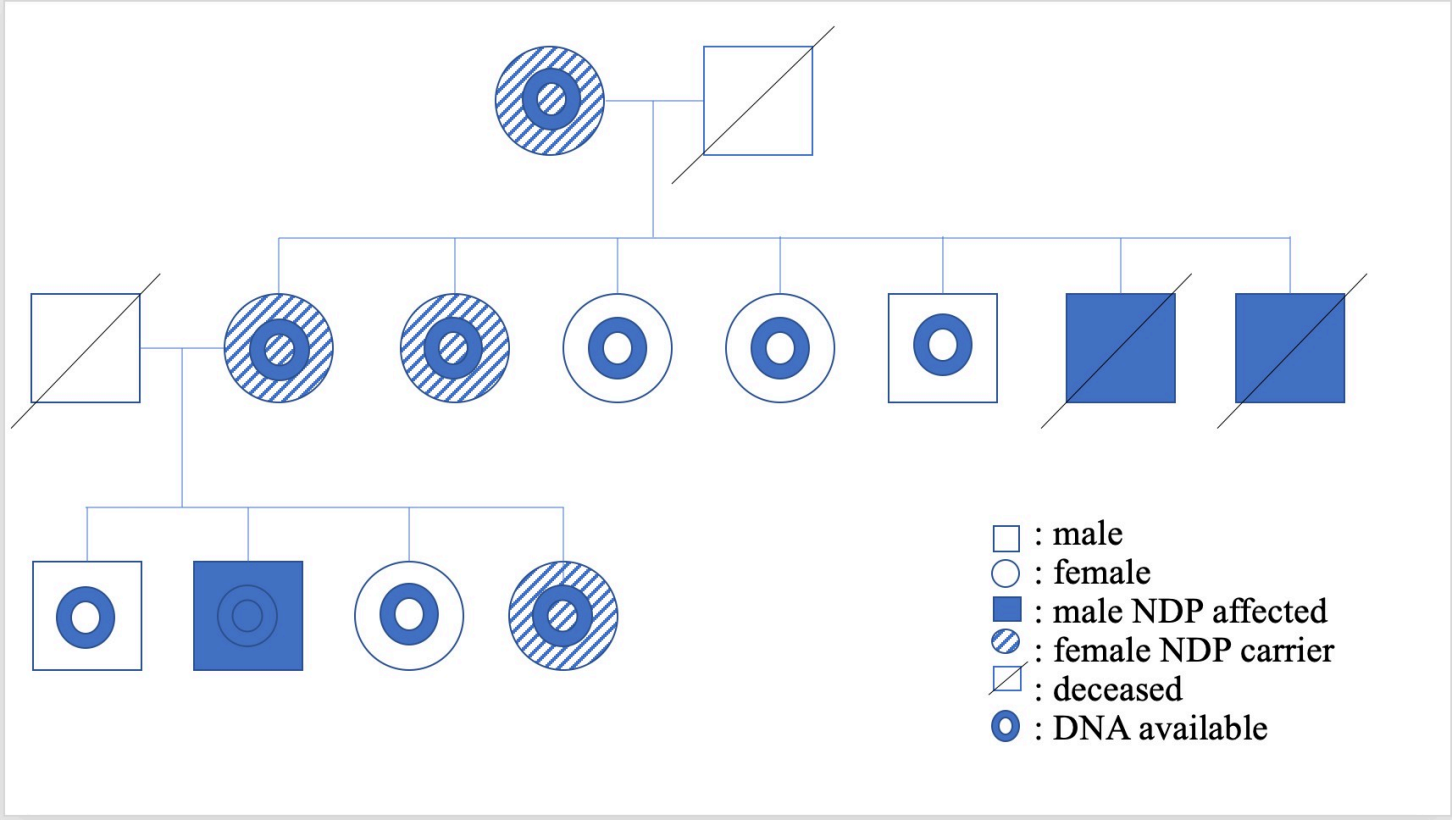
Pedigree of RD affected and related ECS. RD-affected and obligate carriers (sires and dams of the RD-affected ECS), and the dog(s) from which DNA was available are indicated in the legend. Segregation is consistent with an X-linked autosomal recessive mode of inheritance.

Ocular examinations were performed on all twelve related ECS, between 2010–2018, at the Animal Health Trust (AHT) veterinary hospital’s ophthalmology department, by veterinary ophthalmologists, due to owners’ concerns of visual impairment in three of the males. The original two affected males were examined at 6 weeks old in 2010, whilst the affected case was examined at 6weeks, 12 weeks and 12 months old in 2017–18. Five generation pedigrees were obtained for all related ECS (Fig 1). Ocular ultrasonography was performed in the three affected males. The sire of the second litter had been examined for hereditary eye diseases as part of the British Veterinary Associated/Kennel Club/International Sheep Dog Society Eye Scheme.

### Ocular examination

All ophthalmic examinations performed in the study included: vision assessment via maze testing and visual responses; rebound tonometry (TonoVetTM iCare, FI-01510 Vantaa, Finland); slit-lamp biomicroscopy (Kowa SL-17, Torrance, California, USA); and direct and indirect ophthalmoscopy (Welch Allyn Ltd, Ashby de la Zouch, Leicestershire, UK and Keeler Professional, Windsor, Surrey, UK) after dilation with 1% tropicamide (Minims, Bausch & Lomb, Kingston-upon-Thames, Surrey, UK). B-mode ocular ultrasound was performed on the three affected ECS (Easoate MyLabONE, 10-18MHz 30mm linear probe, Imotek, Somersham, Cambs, UK). Topical anaesthetic 0.5% proxymetacaine (Minims, Bausch & Lomb, Kingston-upon-Thames, Surrey, UK) was instilled in the eyes prior to ocular ultrasonography. Carbomer gel (Viscotears, Bausch & Lomb, Kingston-upon-Thames, Surrey, UK) was used as a coupling medium for ocular ultrasonography, and the probe was placed in a horizontal position in contact with the central cornea and axial eye length was measured from the corneal endothelium to the internal sclera. Fundus photography was performed in four ECS (RetCam Shuttle, Clarity, Macclesfield, Cheshire, UK).

### DNA extraction

DNA was extracted from the buccal mucosal swabs using the QIAamp DNA Blood Mini Kit (Qiagen, Manchester, UK) according to the manufacturer’s instructions. DNA concentration and purity were determined by using the NanoDrop 1000 spectrophotometer (Thermo Fisher Scientific, Loughborough, UK) and/or the Qubit fluorometer with the Qubit dsDNA broad range (BR) Assay Kit (Invitrogen, Loughborough, UK). Initial primers for the candidate gene approach were designed using Primer3 and purchased from Integrated DNA Technologies (IDT, Leuven, Belgium) [30].

### Whole genome sequencing

WGS of DNA from one RD-affected ECS was outsourced to Edinburgh Genomics, UK. Illumina sequencing of a TruSeq Nano library on a HiSeq X sequencing platform and paired-end sequencing generated with read lengths of 150 base pairs and approximately 30x coverage of the dog genome. Reads were then aligned to the canine reference genome (CanFam3.1) using the Burrows-Wheeler Aligner and variant calls were made using GATK (HaplotypeCaller) [31, 32]. The WGS was also visualised and manually interrogated using Integrative Genomics Viewer (IGV) [33].WGS data from this dog is available from the European Nucleotide Archive (ENA, Accession number: PRJEB39869).

### Candidate gene approach

Five candidate genes that have been associated with retinal detachment in dogs or X-linked retinal detachments in humans were selected (*NHEJ1*, *COL9A3*, *COL9A2*, *RS1* and *NDP*) [7, 8, 11, 18, 34]. Using IGV, the exons and introns of each candidate gene and 5000bp of flanking DNA were examined for single nucleotide variants (SNVs), insertions and deletions using WGS from an unaffected American cocker spaniel and the CanFam3.1 for reference and comparison. The potential variants were filtered by consideration of their genomic context (i.e. if they are within coding or exonic sequence, or are splice site variants), their presence in the control genomes, their location with respect to repetitive regions and their mapping quality score. Ensembl genome browser, PolyPhen and Provean were used to further analyse potential causal variants to predict pathogenicity [35–37]. PredictProtein and Swiss-Model were used to assess structural differences between the normal and altered NDP protein [38, 39].

### Whole genome approach

WGS from 198 dogs were processed through the same analysis pipeline as the ECS case. Genomic Variant Call Format (VCF) files from 199 canine WGS (198 “controls” and one ECS case) were combined using HaplotypeCaller into a multi-sample VCF file. For cross-genome analysis, Variant Effect Predictor (VEP) was run on the multi-sample file. Two greyhounds were affected with a similar phenotype and were therefore omitted from the multi-sample file. Variants predicted to result in premature start/stop codons, splicing variants, nonsense variants, coding insertions or deletions, or missense variants predicted by Polyphen and Provean to be pathogenic were retained for further investigation [35, 37]. The remaining variants were then filtered based on appropriate segregation (homozygous for the risk-variant in the case and homozygous wild type or heterozygous for the risk variant in the controls). The DBVDC genome dataset containing 616 additional canids was used for additional variant filtering based on appropriate segregation. Pubmed, OMIM, Retnet and Varelect were used to investigate gene function, expression and phenotype of remaining variants [40–42].

### Candidate variant genotyping

Candidate variants that remained after filtering were investigated further by Sanger sequencing of the 10 related ECS (1 affected and 9 unaffected ECS) and appropriate segregation for an autosomal recessive or X-linked mode of inheritance was assessed (S1 Appendix).

The remaining candidate variant in *NDP* was genotyped in 170 ECS and 264 dogs of 93 breeds, using allele specific probes in an allelic discrimination assay. Custom primers designed to be 135 base pairs in length (forward: 5’-GGAGAGGATGTACCGGTAGGT; reverse: ACTTCGGCTGCGCTGTT) and allele-specific probes (variant: FAM-CCTCATGCCCCCCCG; reference: VIC-AGCCTCATGCCCCCCG) (Thermo Fisher Scientific, Loughborough, UK) were used. Reactions were carried out in 8 μL volumes including 4 μL LUNA Universal qPCR Master Mix (New England Biolabs), 1.4 μL MQ, 0.4 μL DMSO, 0.2 μL of primers and 2 μL genomic DNA. Cycling parameters were 25°C for 30 seconds, 95°C for 3 minutes, 40 cycles of 95°C for 3 seconds and 60°C for 10 seconds, and finally 25°C for 30 seconds.

### Statistics

Using Microsoft Excel, a Fisher’s exact test using a 2 x 2 contingency table was used to assess significance of association of the candidate variant with RD using the one affected and 170 unaffected ECS. Statistical significance was set at P ≤ 0.05.

## Results

### Clinical findings

The related ECS were all purebred dogs that resided in the UK. A pedigree was established of those related ECS (Fig 1). In the original litter examined (seven puppies), two males were found to have behavioural signs suggestive of severe visual deficits (determined by observing their movements in unfamiliar surroundings), absent pupillary light reflexes, wandering nystagmus, darkened irises, and extensive posterior segment haemorrhage. Ocular ultrasonography revealed total retinal detachment and increased echogenicity within posterior segment in all eyes. The globe diameters (anterior to posterior) were comparable with their normal litter mates at 16mm in all dogs except one affected dog, whose globes were 15mm OU. Both males were euthanised, one dog when less than one year old due to behavioural abnormalities and blindness, the other at six years old due to suspected glaucoma and aggression. One male was found to be bilaterally cryptorchid on clinical examination.

**Fig 2:**
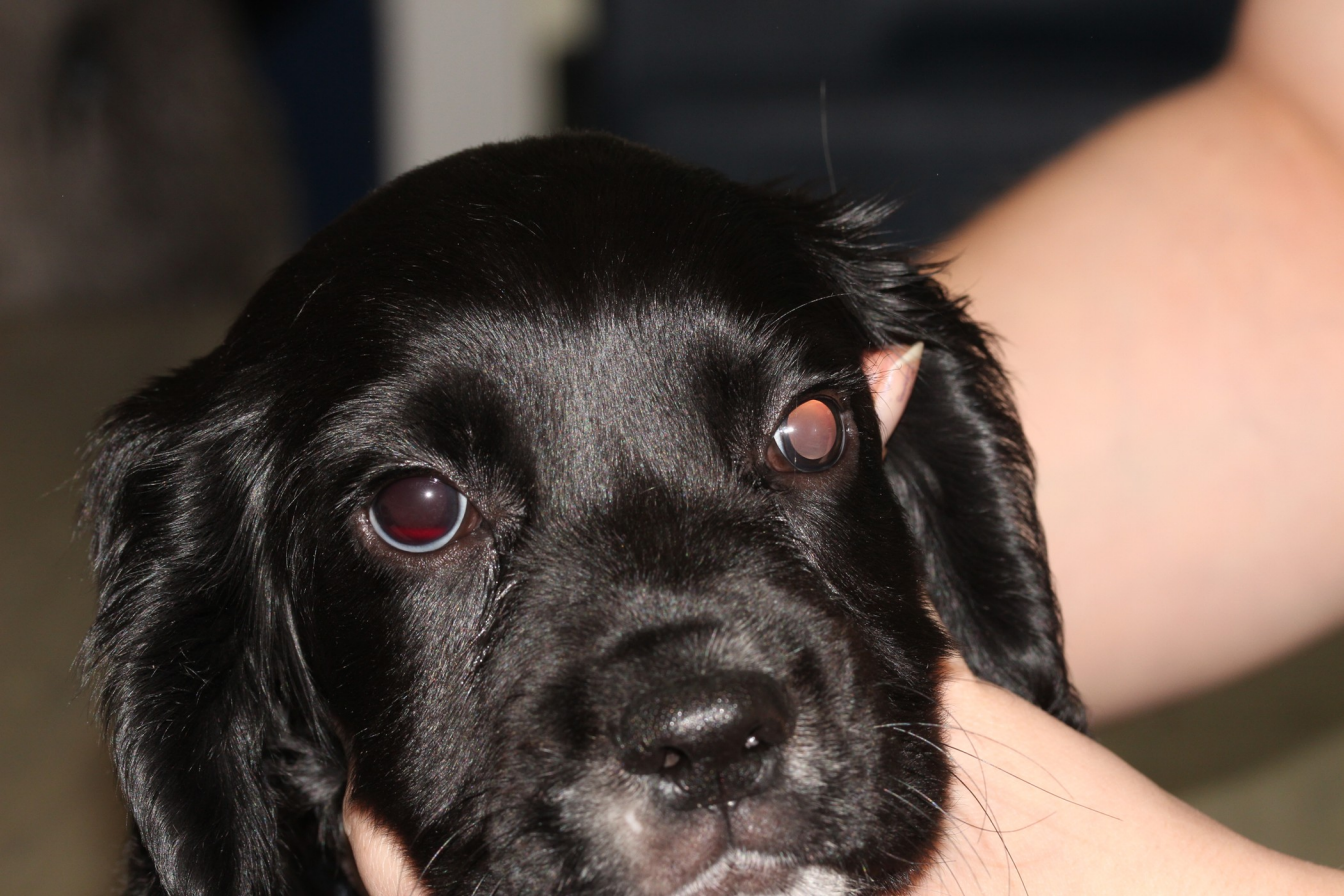
Ocular defects associated with affected ECS. Showing RD-affected male with hyphema of the right eye.

**Fig 3:**
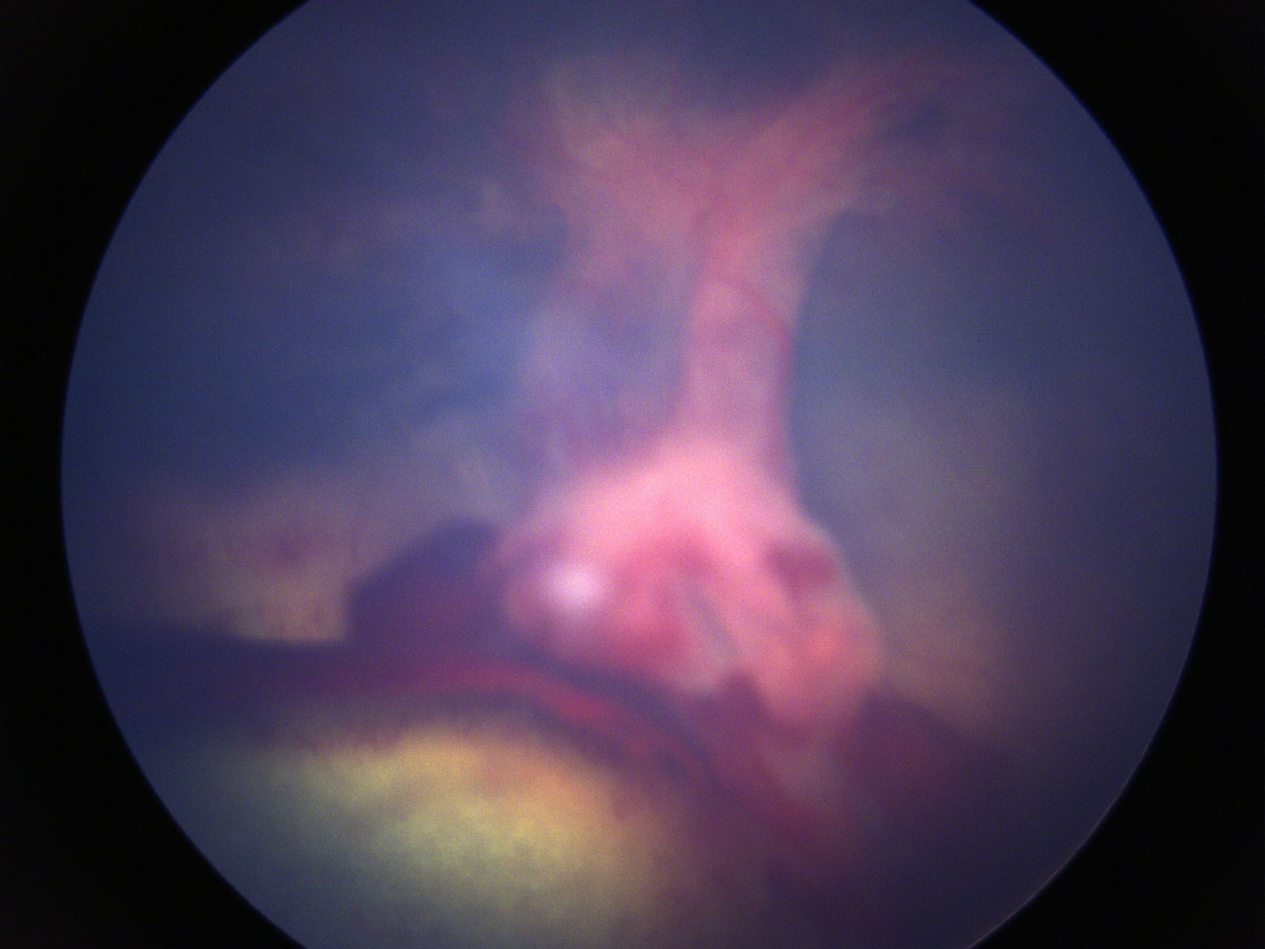
Ocular defects associated with affected ECS. Showing the retinal detachment as a fibrovascular mass.

**Fig 4:**
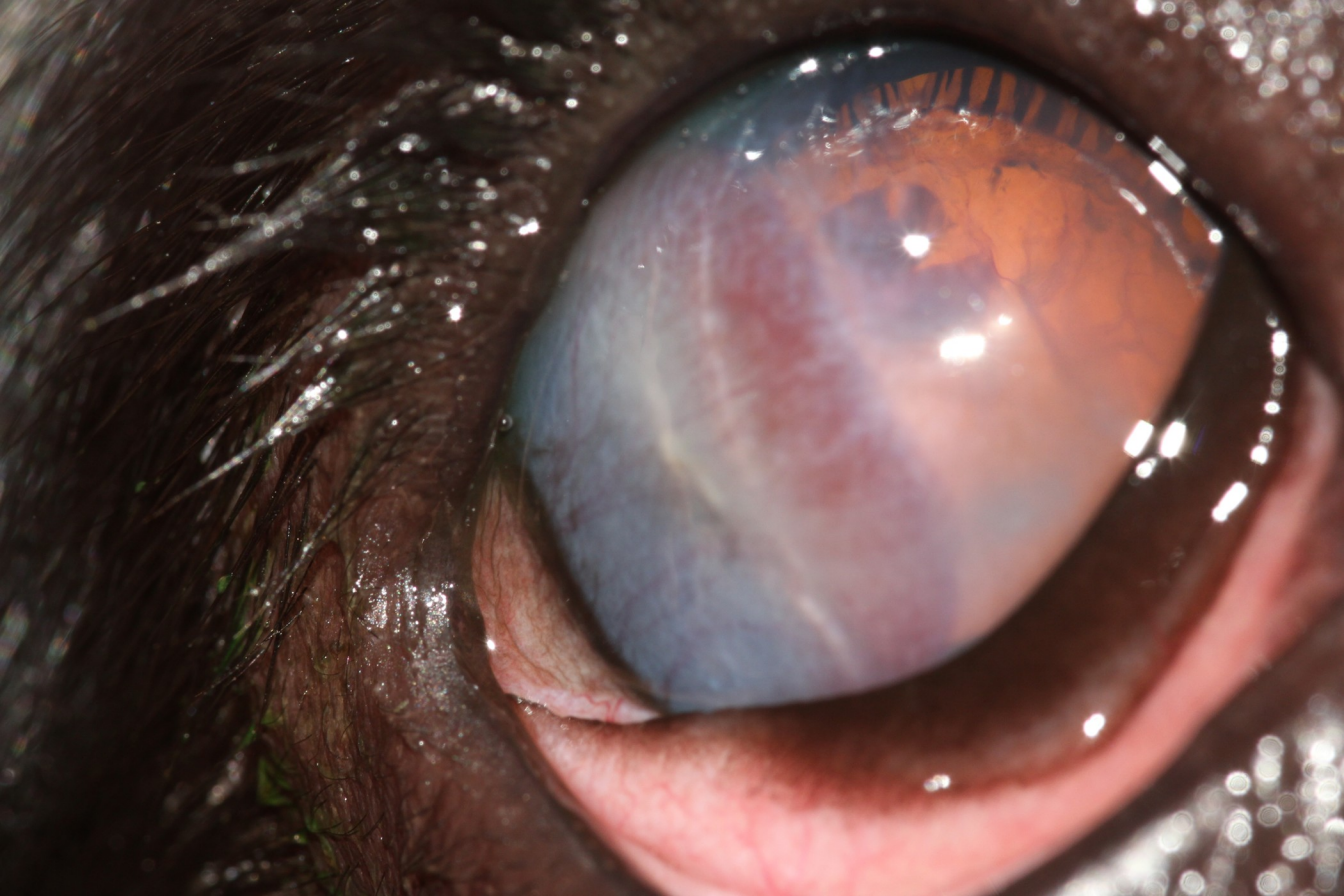
Ocular defects associated with affected ECS. Showing corneal and lenticular abnormalities.

**Fig 5:**
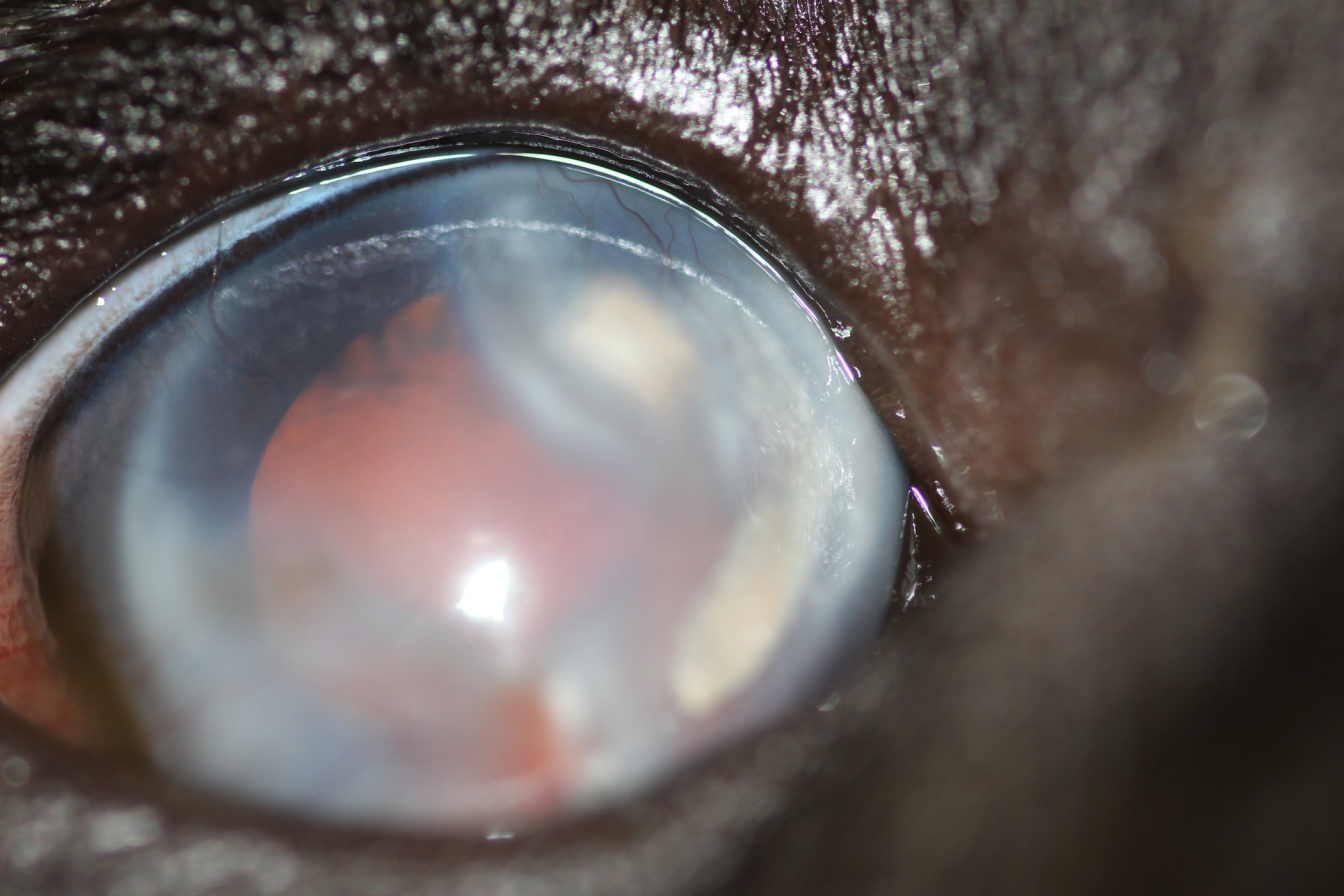
Ocular defects associated with affected ECS. Showing corneal and lenticular abnormalities.

The sire of the second litter had no abnormalities detected on ocular examination. In the second litter examined (four puppies), one male displayed behavioural signs suggestive of severe visual deficits (determined by observing his movements in unfamiliar surroundings), and was found to have absent dazzle and pupillary light reflexes, a wandering nystagmus, corneal endothelial opacities ventrally in the right eye, darkened irises, hyphema of the right eye and extensive posterior segment haemorrhage in both eyes, with the retina in the left eye appearing detached and as a fibrovascular retrolental mass (Figs 2 and 3). Ocular ultrasonography revealed total retinal detachment and increased echogenicity within the posterior segment in both eyes. The left globe appeared microphthalmic with an anterior-posterior diameter of 16.42mm versus 21.88mm in the right eye. The affected male was subsequently re-examined, the left eye appeared relatively unchanged whereas the corneal pathology had advanced to diffuse corneal degeneration with neovascularisation and dense crystalline stromal deposits in the right eye (Fig 4). Microphakia was evident along with incipient nuclear cataract being present (Fig 5). On clinical examination the dog was found to be cryptorchid at 10 months old.

### Identification of candidate variants by a candidate gene approach

**Fig 6:**
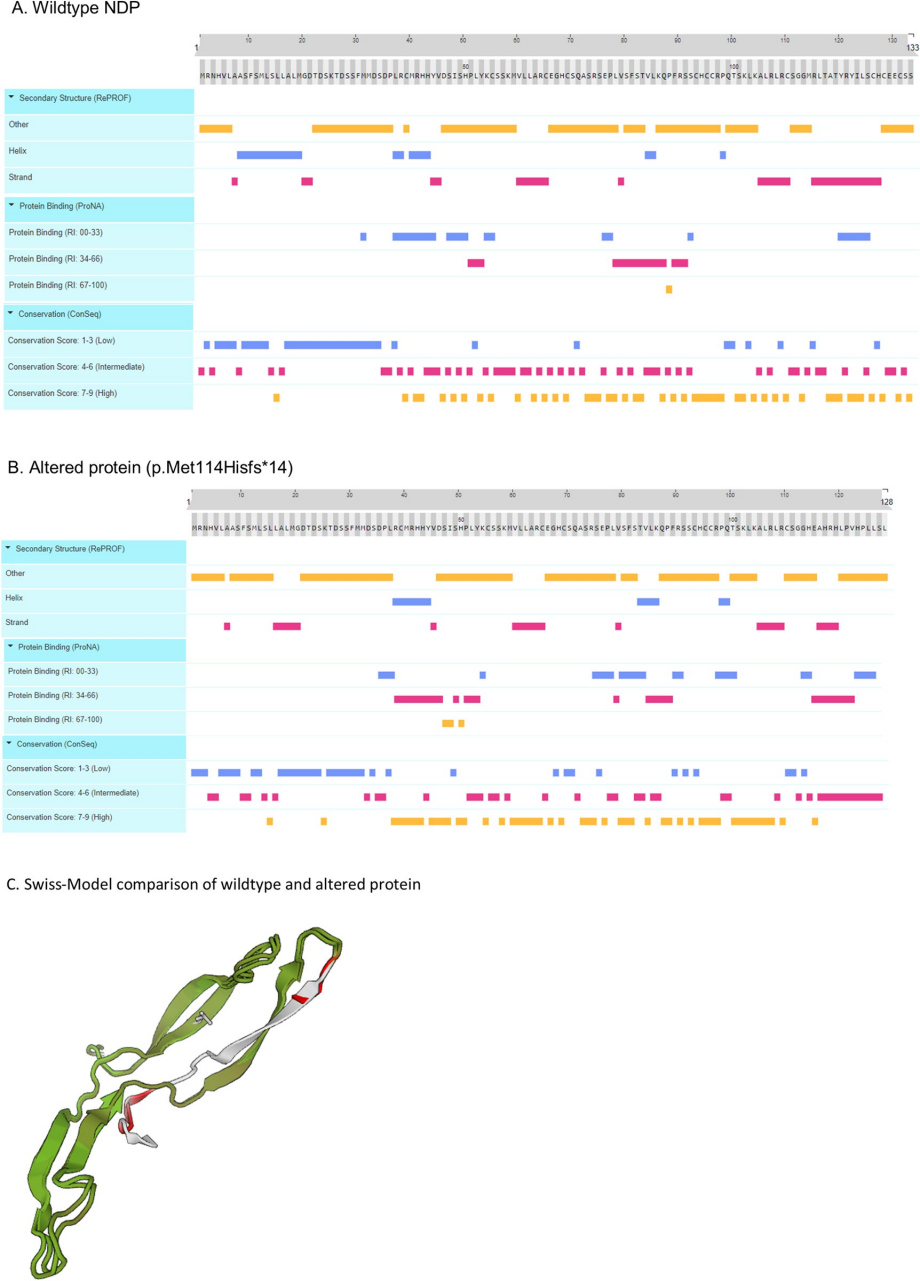
Comparison of the wildtype (A) and altered protein (B). Using PredictProtein reveal that the two proteins are expected to differ throughout with regards to secondary structure, protein binding and conservation. In addition, modelling of both proteins using Swiss-Model (C) reveal that in the altered molecule, the 15 amino acids at the C-terminus, involved in the shifted reading frame, do not align with the N-terminus (white and red in C) of the wildtype protein at all, and also do not align with any other known protein structures.

Manual visual inspection of the DNA sequence of the five candidate genes, comparing the affected ECS with 1 unaffected dog and the CanFam3.1 reference using IGV, identified 196 variants that were initially narrowed down to 12 potential variants by the manual interrogation described above. This was then further narrowed down on the basis of predicted pathogenicity (using Provean and Polyphen-2) to two potential variants. The first was a missense variant in exon seven in *COL9A3* (c.1240G>C, p.Asp414His), predicted to be “probably damaging” by PolyPhen and “neutral” by Provean. The second was a frameshift variant in *NDP* (c.338_339insC), that was predicted to result in 15 aberrant amino acids before a premature stop in norrin protein (p.Met114Hisfs*16), resulting in a loss of the last five amino acids. Each of the aberrant amino acids was independently assessed for pathogenicity using PolyPhen-2 and Provean (Table): four changes were predicted to be “Probably Damaging” by PolyPhen-2 and nine changes were predicted to be “Deleterious” by Provean (Table 1). The NDP protein is made up of 133 amino acids; therefore, 15.0% of the protein is predicted to be either changed or lost. In addition, significant structural differences between the wildtype and altered proteins are predicted (Fig 6).

**Table 1:** Predicted pathogenicity.

Amino acid change	PolyPhen Result	PolyPhen Score	Provean Result	Provean Score (Threshold <-2.5)
M114H	Possibly Damaging	0.566	Neutral	-0.102
R115E	Possibly Damaging	0.462	Neutral	-0.647
L116A	Possibly Damaging	0.816	Deleterious	-3.109
T117H	Probably Damaging	0.974	Deleterious	-3.353
A118R	Probably Damaging	0.958	Neutral	-1.118
T119H	Probably Damaging	0.974	Neutral	-1.647
Y120L	Possibly Damaging	0.816	Deleterious	-8
R121P	Possibly Damaging	0.827	Deleterious	-4.941
Y122V	Possibly Damaging	0.816	Deleterious	-6.235
I123H	Possibly Damaging	0.944	Deleterious	-6.882
L124P	Probably Damaging	0.974	Neutral	-1.588
S125L	Possibly Damaging	0.633	Deleterious	-3.176
C126L	Possibly Damaging	0.462	Deleterious	-10
H127S	Possibly Damaging	0.462	Neutral	2.255
C128L	Possibly Damaging	0.462	Deleterious	-7.118

### Identification of candidate variants by a whole genome sequencing approach

WGS data from the RD-affected ECS was compared to those from 198 unaffected control dogs of 98 other breeds. There was a total of 27,874,423 variants in at least one of these genomes. These variants were first filtered based on predicted effect on a protein using the Ensembl Variant Effect Predictor (VEP) [43, 44], resulting in 530 variants. These variants were further filtered based on appropriate segregation for an autosomal recessive mode of inheritance which resulted in 23 remaining variants (Table 2).

**Table 2:** 23 remaining variants.

Chromosome	Genomic coordinates	Reference allele	Alternate allele	Consequence	Gene Symbol	Gene Full Name	Human Orthologue	Present in DBVDC	Base change	Amino acid change	CDS Position	Protein Position
8	33957623	A	C	missense_variant	DACT1	Dishevelled Binding Antagonist of Beta Catenin 1	DACT1	Yes	gAg/gCg	E/A	1700	567
25	35087169	C	G	missense_variant	BMP1	Bone Morphogenetic Protein 1	BMP1	Yes	gaG/gaC	E/D	114	38
26	9447613	TA	T,TTCTTCTGGTAAA,TTCTTCTGGTAA	frameshift_variant	ENSCAFG00000008764		None	Yes	Atc/tc	I/X	1549	517
38	21369593	G	GT	frameshift_variant	KLHDC9	kelch domain containing 9	KLHDC9	No	-/A	-/X	735–736	245–246
31	39447278	G	A	missense_variant	COL6A2	Collagen Type IX Alpha 2 Chain	COL6A2	Yes	aGa/aAt	S/N	2495	832
17	20301410	G	GC	frameshift_variant	GAREM2	Grb2-associated regulator of Erk/MAPK1	GAREM2	No	ggg/ggCg	G/GX	620–621	207
18	32393127	CGCCGCCTCCAGCCACGCGGCCGCCTGCGCCTCCGAGAAGCCGCGGGGCACCCGCTCCTCCAG	C	frameshift_variant	FJX1	Four Jointed Box Kinase 1	FJX1	No	CTGGAGGAGCGGGTGCCCCGCGGCTTCTCGGAGGCGCAGGCGGCCGCGTGGCTGGAGGCGGCg/g	LEERVPRGFSEAQAAAWLEAA/X	199–260	67–87
6	8811462	ATCTGCTCGTATCTGGAGATGCTGTTAGAGTGCTGG	*,GTCTGCTCGTATCTGGAGATGCTGTTAGAGTGCTGG	missense_variant	ENSCAFG00000014044		None	Yes	aCCAGCACTCTAACAGCATCTCCAGATACGAGCAGATcc/aCCAGCACTCTAACAGCATCTCCAGATACGAGCAGACcc	TSTLTASPDTSRS/TSTLTASPDTSRP	7454–7489	2485–2497
12	52207701	GAAAGAAAGAAAGAAAA	*,GAAAGAAGAAAAAGAAAGAAAGAAAA,G,GAAAAGAAAGAAAGAAAA,GAAGAAAGAAAGAAAA	inframe_insertion+frameshift_variant	ENSCAFG00000030881+ENSCAFG00000030881		None	Yes	tTTTTCTTTCTTTCTTTc/tTTTTCTTTCTTTCTTTTTCTTCTTTc tTTTTCTTTCTTTCTTTc/tc	FFFLSF/FFFLSFSSF FFFLSF/X	203–218+203–218	68–73+68–73
1	77496026	CTTTCTTCTTTCTCTTTCTTTCTTTCTTTCTTTCTTTCTTTCTTTCTTCTTTCTCTTTCTTTCTTTCTTTCTTTCTTTCTTTCTTTTCTTTCTTTCTTTCTTTCTTTCTTTCTTTCTTTCT	CTCTTCTTTCTCTTTCTTTCTTTCTTTCTTTCTTTCTTTCTTTCTTCTTTCTCTTTCTTTCTTTCTTTCTTTCTTTCTTTCTTTTCTTTCTTTCTTTCTTTCTTTCTTTCTTTCTTTCT,C,*,CTTTTTTCTTCTTTCTCTTTCTTTCTTTCTTTCTTTCTTTCTTTCTTTCTTCTTTCTCTTTCTTTCTTTCTTTCTTTCTTTCTTTCTTTTCTTTCTTTCTTTCTTTCTTTCTTTCTTTCTTTCT	splice_acceptor_variant&intron_variant&non_coding_transcript_variant	ENSCAFG00000034426		None	Yes				
6	55325921	AAG	*,A	splice_acceptor_variant&intron_variant	ENSCAFG00000032258		None	Yes				
6	54025399	G	GAAA,GA,A,*,GAAAA,GAAAGAAAA	inframe_insertion+frameshift_variant+missense_variant	ENSCAFG00000029585+ENSCAFG00000029585+ENSCAFG00000029585		None	Yes	aGg/aGAAAg aGg/aGAg aGg/aAg	R/RK R/RX R/K	77+77+77	26+26+26
16	12395809	A	G,*	missense_variant	ENSCAFG00000031012		None	Yes	aAa/aGa	K/R	422	141
1	77496030	CTTCTTTCTCTTTCTTTCTTTCTTTCTTTCTTTCTTTCTTTCTTCTTTCTCTTTCTTTCTTTCTTTCTTTCTTTCTTTCTTTTCTTTCTTTCTTTCTTTCTTTCTTTCTTTCTTTCT	C,*,CTTTCTTTCTCTTTCTTTCTTTCTTTCTTTCTTTCTTTCTTTCTTCTTTCTCTTTCTTTCTTTCTTTCTTTCTTTCTTTCTTTTCTTTCTTTCTTTCTTTCTTTCTTTCTTTCTTTCT,CTCTTTCTCTTTCTTTCTTTCTTTCTTTCTTTCTTTCTTTCTTCTTTCTCTTTCTTTCTTTCTTTCTTTCTTTCTTTCTTTTCTTTCTTTCTTTCTTTCTTTCTTTCTTTCTTTCT,CTCTTTCTTTCTCTTTCTTTCTTTCTTTCTTTCTTTCTTTCTTTCTTCTTTCTCTTTCTTTCTTTCTTTCTTTCTTTCTTTCTTTTCTTTCTTTCTTTCTTTCTTTCTTTCTTTCTTTCT	splice_acceptor_variant&intron_variant&non_coding_transcript_variant	ENSCAFG00000034426		None	Yes				
11	54593727	C	G	missense_variant	ENSCAFG00000031828		None	No	Gac/Cac	D/H	148	50
21	4789963	T	G	missense_variant	CCDC82	Coiled-coil Domain Containing 82	CCDC82	Yes	gaT/gaG	D/E	171	57
25	44173062	G	A	missense_variant	CHRND	Cholinergic Receptor Nicotinic Delta Subunit	CHRND	No	cGc/cAc	R/H	1400	467
34	19179708	G	A	missense_variant	TBCCD1	TBCC Domain Containing 1	TBCCD1	No	aCg/aTg	T/M	167	56
39	1615231	G	A	missense_variant&splice_region_variant	ARSF	Arylsulfatase F	ARSF	No	aGg/aAg	R/K	161	54
39	37950668	G	GC	frameshift_variant	NDP	Norrin Cystine Knot Growth Factor	NDP	No	ggc/ggGc	G/GX	338–339	113
6	55887792	C	G	missense_variant	CCDC18	Coiled-Coil Domain Containing 18	CCDC18	Yes	atG/atC	M/I	1230	410
7	9830091	C	A	missense_variant	TRAF5	TNF Receptor Associated Factor 5	TRAF5	No	Caa/Aaa	Q/K	1003	335
9	59422620	G	A	missense_variant	DENND1A	DENN Domain Containing 1A	DENND1A	No	cGc/cAc	R/H	2279	760

Two remaining variants FJX1 and NDP involved in retinal development highlighted in blue.

Of the 23 variants only 14 were within known genes that had human orthologues. Five of these variants were found in the additional 648 canid genomes within the DBVDC and were excluded from further investigation. This resulted in 9 variants in 9 individual genes. Gene function was investigated using Pubmed, OMIM, Retnet and Varelect. Only two of these genes were known to be involved in retinal development: *NDP* and *FJX1* (four jointed box kinase 1). The variant in *NDP* was identical to that discovered by the candidate gene approach (c.338_339insC). The variant in *FJX1* was a frameshift variant (c.199_260del).

### Genotyping the candidate variants

Sanger sequencing was used to genotype the nine related ECS and the one RD-affected dog for which DNA was available. The RD-affected dog was homozygous for two of the variant alleles (*NDP*, *COL9A3*) but was homozygous for the wildtype *FJX1* allele with sanger sequencing. Manual inspection of the *FJX1* variant region in the affected WGS revealed highly repetitive sequence and reads with poor alignment with CanFam3.1, and the calling of this as a variant is therefore, a false positive. Three unaffected ECS were also homozygous for the *COL9A3* variant, whereas all nine unaffected dogs were either heterozygous or homozygous for the wildtype *NDP* allele and all nine unaffected dogs were homozygous wild type for *FJX1*. Therefore, only the *NDP* variant segregated appropriately for an autosomal recessive or X-linked mode of inheritance among the nine related ECS (Fig 6). An allelic discrimination assay was then performed in the additional 170 unaffected ECS and 264 unaffected dogs of 93 other breeds. All these dogs were homozygous for the wild type allele. Association of the *NDP* variant with RD was significant (P = 0.0056).

## Discussion

In this study, two different approaches, candidate-gene and whole genome, were used to identify a single exonic insertion variant in *NDP* which was shown to be associated with X-linked RD in the ECS. This variant is predicted to result in a 15 residue shift in the reading frame, a premature stop codon and norrin protein truncated by five residues, out of a normal length of 133 amino acids. While structural variants near exons would have been detected in the candidate gene approach, our current whole genome analysis pipeline does not detect structural variants. It is, therefore, worth noting that the presence of structural variation causing disease in this study cannot be fully excluded. In addition, the canine genome is not well annotated with regulatory regions and lncRNAs, and these were therefore not investigated and cannot be excluded. Indeed, a lncRNA (NDP-AS1) on the reverse strand is annotated on the human genome build GRCh38.p13), but it is unclear if the canine genome shares this feature, and if it does whether the variant described here affects the NDP protein, the lncRNA or both. It is also worth noting linkage disequilibrium (LD) was not investigated due the small number of WGS available for ECS in this study (four in total including the case), which was considered too few samples for an accurate estimate of LD in the breed in general and especially in the vicinity of the variant of interest by the authors.

This is the first time a variant in *NDP* has been described in the dog and the first time X-linked RD has been reported in the dog. The results suggest that this variant is likely to be private to this family of ECS as it was not identified in the additional 170 ECS and the 1078 other non-ECS canids. The pedigree analysis was helpful in determining it was most likely a recessive condition as only a small proportion of the progeny was affected and, because it was only observed in male progeny, a X-linked mode of inheritance was also hypothesized. This narrowed down the potential candidate genes to be investigated. Genomic material was only available from one affected as the other two affected were deceased. The low number of affected animals is a limiting factor for this study. Ocular tissue from RD-affected ECS would also be helpful for assessing changes in the mRNA and protein expression within the retina of these dogs in comparison to age matched control dog retinas. Further work is required to prove this is the causal variants of RD in the ECS, although the predicted effect on the protein is supportive of this hypothesis.

WGS is a very useful and cost-effective method of identifying variants, when faced with a small number of affected cases. Unlike genome wide association studies, a small number, or even a single case may be sufficient to identify underlying variant as in this study. If successful, WGS can help identify risk variants early on in the disease emergence process, hence eliminating it before the disease becomes widespread within the population, which is especially important in dogs due to high levels of inbreeding, popular sire effects and relatively fast turnover of progeny [45, 46]. A limitation to using WGS as with other sequencing techniques, is the inherent difficulty in the detection of structural variants, for example, transposons, inversions, or large insertions and deletions which our current WGS pipeline cannot identify. Genome-wide association studies can be useful in identifying the approximate location of the disease variant even when there is a large structural variant, so its use in combination with WGS could help mitigate this limitation. Unfortunately, due to our low case numbers, this was not possible in this study. It is therefore worth noting that the presence of structural variation causing disease cannot be fully excluded.

The candidate gene approach was considered appropriate for this study because similar phenotypes had been reported in humans and dogs which have been shown to be associated with a limited number of genes. This approach is useful when trying to identify the genetic basis of a disease thought to be of simple inheritance using only a small number of cases [47]. It requires existing knowledge about the known or presumed biology of the phenotype under investigation, either in other breeds of dog or in other species, which was available in this instance [48]. This method is however biased and limited, as it only includes genes known to be associated with the relevant phenotype, meaning variants in novel genes not previously associated with the phenotype will go undetected [49]. A whole genome approach was also used, which does not require prior knowledge of gene candidacy and thus is considered a less biased approach compared to candidate gene analysis [50].

*NDP* encodes for norrin, a cysteine knot growth factor, that is required for angiogenesis in the eye, ear, brain and female reproductive system [47]. Norrin is an atypical Wnt (wingless-related integration site) ligand, that has binding sites for both *Fz4* (Frizzled 4) and *LRP5/6* (low-density lipoprotein receptor-related protein) which activates β-catenin signalling, that plays a central role in retinal vascularisation [47, 51]. Its structure is a semi-circular shaped homodimer linked through three intermolecular disulphide bonds. Disease-causing variants will affect the protein folding and stabilisation of the norrin structure and hence affect protein function [52].

Over 100 disease-causing *NDP* variants have been reported in humans [26]. Analysis of gnomAD data within the equivalent human C-terminal region affected by the frameshift and truncation (X:43809045–43809107, based on genome build GRCh37/hg19) revealed eight variants. Of these, three are missense, five are synonymous and none are truncating. However, analysis of the ClinVar data in gnomAD revealed five additional “likely pathogenic” variants, including three missense and two nonsense/stop gained variants [53]. These variants can result in inherited retinopathies including Norrie disease, X-linked familial exudative vitreoretinopathy, retinopathy of prematurity and Coat’s disease [54]. Retinal detachment has been reported in all of these conditions to varying degrees at different clinical stages [55–57]. Retinal detachment in Norrie disease has even been reported in utero during the third trimester in a known carrier of the condition [58]. The affected ECS males were examined at 6 weeks old, although blindness was suspected earlier than this. Puppies open their eyes at approximately two weeks of age [59]. In the affected ECS, retinal detachment was suspected to be congenital, but this cannot be confirmed.

Histopathological characterisation is usually not possible in humans with *NDP*-related retinopathies. Therefore *Ndph* (Norrie disease homologue)-knockout mice have been used as a small animal model to study early histopathological changes [60]. In mice with targeted inactivation of *Ndp*, histological data suggest retrolental structures and a disorganized ganglion cell layer as primary pathogenic events [61]. Histopathology of the retina and posterior segment of an RD-affected ECS would be useful to better identify the phenotypic characteristics of this type of RD in the dog. No ocular tissue was available from the affected dog in this study, so the pathogenesis can only be hypothesised based on the clinical findings observed. Although there were subtle ophthalmic differences in the three affected dogs, the overall ocular changes were similar, as they all shared the congenital blindness, extensive posterior segment haemorrhage and the retinal detachment. The congenital retinal detachments in the form of fibrovascular retrolental masses and extensive vitreal haemorrhage, is more similar to that described in clinical cases of human Norrie disease than the other inherited retinopathies associated with *NDP* variants [62, 63].

Cognitive impairment, behavioural disturbances and progressive sensorineural hearing loss have been described in humans with Norrie disease. The two affected males of the initial litter of ECS were euthanised early in life due to behavioural abnormalities that were thought to be related to their lack of vision. The affected male in the second litter was not reported to show behavioural abnormalities. No males were reported to have any hearing impairment, although brainstem auditory evoked response testing was not performed to confirm this, in the affected ECS to confirm this. Hearing loss is reported to develop in humans in their early twenties so it is possible that the affected males had not yet reached an appropriate age for this to have developed and the one surviving affected male could develop hearing loss as he continues to mature [22].

This is the first time a variant in *NDP* has been associated with a retinal disease in the dog and also the first X-linked RD to be reported in the dog. Therefore, this study also suggests the dog may serve as a useful model for understanding human *NDP*-related retinopathies and the development of gene and other therapies.

## Supporting information

S1 Appendix Reaction mixes and thermal cycling parameters: Sanger sequencing of candidate variants. (DOCX)

## References

[1] Iwabe S, Dufour VL, Guzmán JM, Holle DM, Cohen JA, Beltran WA, et al. Focal/multifocal and geographic retinal dysplasia in the dog—In vivo retinal microanatomy analyses. Veterinary Ophthalmology. 2020 3;23(2):292–304. 10.1111/vop.12725 31746146 PMC7071990

[2] Narfström K and Petersen-Jones S. Diseases of the canine ocular findus. In: Gelatt KN, Gilger BC, Kern TJ. Veterinary Ophthalmology. John Wiley & Sons; 2012. Pp. 1316–1320.

[3] Rubin LF. Heredity of retinal dysplasia in Bedlington terriers. Journal of the American Veterinary Medical Association. 1968 1 1;152(3):260.

[4] Ashton N, Barnett KC, Sachs DD. Retinal dysplasia in the Sealyham terrier. The Journal of Pathology and Bacteriology. 1968 10;96(2):269–72. 10.1002/path.1700960203 5748920

[5] Carrig CB, MacMillan A, Brundage S, Pool RR, Morgan JP. Retinal dysplasia associated with skeletal abnormalities in Labrador Retrievers. Journal of the American Veterinary Medical Association. 1977 1;170(1):49–57. 830631

[6] Meyers VN, Jezyk PF, Aguirre GD, Patterson DF, Short-Limbed dwarfism and ocular defects in the Samoyed dog. J Am Vet Med Assoc. 1983; 183(9):975–9 Epub 1983/11/01. 12002589

[7] Stavinohova R, Hartley C, Burmeister LM, Ricketts SL, Pettitt L, Pont RT, et al. Clinical, histopathological and genetic characterisation of oculoskeletal dysplasia in the Northern Inuit Dog. PLOS ONE. 2019;14(8). 10.1371/journal.pone.0220761 31415586 PMC6695176

[8] Goldstein O, Guyon R, Kukekova A, Kuznetsova TN, Pearce-Kelling SE, Johnson J, et al. COL9A2 and COL9A3 mutations in canine autosomal recessive oculoskeletal dysplasia. Mammalian Genome. 2010 8 1;21(7–8):398–408. 10.1007/s00335-010-9276-4 20686772 PMC2954766

[9] Barnett KC. Collie eye anomaly (CEA). Journal of Small Animal Practice. 1979 9;20(9):537–42. 10.1111/j.1748-5827.1979.tb06762.x 480920

[10] Lowe JK, Kukekova AV, Kirkness EF, Langlois MC, Aguirre GD, Acland GM, et al. Linkage mapping of the primary disease locus for collie eye anomaly. Genomics. 2003 7 1;82(1):86–95. 10.1016/s0888-7543(03)00078-8 12809679

[11] Parker HG, Kukekova AV, Akey DT, Goldstein O, Kirkness EF, Baysac KC, et al. Breed relationships facilitate fine-mapping studies: a 7.8-kb deletion cosegregates with Collie eye anomaly across multiple dog breeds. Genome research. 2007 11 1;17(11):1562–71. 10.1101/gr.6772807 17916641 PMC2045139

[12] Kropatsch R, Akkad DA, Frank M, Rosenhagen C, Altmüller J, Nürnberg P, et al. A large deletion in RPGR causes XLPRA in Weimaraner dogs. Canine Genetics and Epidemiology. 2016 12 1;3(1):7. 10.1186/s40575-016-0037-x 27398221 PMC4938961

[13] Gurda BL, Bradbury AM, Vite CH. Focus: Comparative Medicine: Canine and feline models of human genetic diseases and their contributions to advancing clinical therapies. The Yale journal of Biology and Medicine. 2017 9;90(3):417. 28955181 PMC5612185

[14] George ND, Yates JR, Moore AT. X linked retinoschisis. The British Journal of Ophthalmology. 1995 7;79(7):697. 10.1136/bjo.79.7.697 7662639 PMC505202

[15] Warburg M. Norrie’s disease. A congenital progressive oculo-acoustico-cerebral degeneration. Acta Ophthalmol (Copenh). 1966;89:1–47. 6013082

[16] Neriyanuri S, Dhandayuthapani S, Arunachalam JP, Raman R. Phenotypic characterization of X-linked retinoschisis: Clinical, electroretinography, and optical coherence tomography variables. Indian Journal of Ophthalmology. 2016 7;64(7):513. 10.4103/0301-4738.190140 27609164 PMC5026077

[17] Pimenides D, George ND, Yates JR, Bradshaw K, Roberts SA, Moore AT, et al. X-linked retinoschisis: clinical phenotype and RS1 genotype in 86 UK patients. Journal of Medical Genetics. 2005 6 1;42(6):e35-. 10.1136/jmg.2004.029769 15937075 PMC1736077

[18] Sauer CG, Gehrig A, Warneke-Wittstock R, Marquardt A, Ewing CC, Gibson A, et al. Positional cloning of the gene associated with X-linked juvenile retinoschisis. Nature Genetics. 1997 10;17(2):164. 10.1038/ng1097-164 9326935

[19] Retinoschisis Consortium. Functional implications of the spectrum of mutations found in 234 cases with X-linked juvenile retinoschisis (XLRS). Human Molecular Genetics. 1998 7 1;7(7):1185–92. 10.1093/hmg/7.7.1185 9618178

[20] Yi J, Li S, Jia X, Xiao X, Wang P, Guo X, et al. Novel RS1 mutations associated with X-linked juvenile retinoschisis. International Journal of Molecular Medicine. 2012 4 1;29(4):644–8. 10.3892/ijmm.2012.882 22245991 PMC3573736

[21] Sims KB. NDP-related retinopathies. InGeneReviews®[Internet] 2014 Sep 18. University of Washington, Seattle. 20301506

[22] Smith SE, Mullen TE, Graham D, Sims KB, Rehm HL. Norrie disease: extraocular clinical manifestations in 56 patients. American Journal of Medical Genetics Part A. 2012 8;158(8):1909–17 10.1002/ajmg.a.35469 22786811

[23] Moreira-Filho CA, Neustein I. A presumptive new variant of Norrie’s disease. Journal of Medical Genetics. 1979 4 1;16(2):125–8. 10.1136/jmg.16.2.125 572429 PMC1012736

[24] Gal A, Wieringa B, Smeets DF, Bleeker-Wagemakers L, Ropers HH. Submicroscopic interstitial deletion of the X chromosome explains a complex genetic syndrome dominated by Norrie disease. Cytogenetic and Genome Research. 1986;42(4):219–24. 10.1159/000132282 3502689

[25] Xu Q, Wang Y, Dabdoub A, Smallwood PM, Williams J, Woods C, et al. Vascular development in the retina and inner ear: control by Norrin and Frizzled-4, a high-affinity ligand-receptor pair. Cell. 2004 3 19;116(6):883–95. 10.1016/s0092-8674(04)00216-8 15035989

[26] Ye X, Wang Y, Nathans J. The Norrin/Frizzled4 signaling pathway in retinal vascular development and disease. Trends in Molecular Medicine. 2010 9 1;16(9):417–25. 10.1016/j.molmed.2010.07.003 20688566 PMC2963063

[27] Rehm HL, Zhang DS, Brown MC, Burgess B, Halpin C, Berger W, et al. Vascular defects and sensorineural deafness in a mouse model of Norrie disease. Journal of Neuroscience. 2002 6 1;22(11):4286–92. 12040033 10.1523/JNEUROSCI.22-11-04286.2002 PMC6758776

[28] Cunliffe J. The encyclopedia of dog breeds, 1st ed, Bath: Parragon; 2000

[29] Jagannathan V, Drögemüller C, Leeb T. Dog Biomedical Variant Database Consortium (DBVDC). A comprehensive biomedical variant catalogue based on whole genome sequences of 582 dogs and 8 wolves. Anim. Genet. 2019;50:695–704. 10.1111/age.12834 31486122 PMC6842318

[30] Rozen S, Skaletsky H. Primer3 on the WWW for general users and for biologist programmers. Methods Mol Biol. 2000; 132:365–86. 10.1385/1-59259-192-2:365 10547847

[31] Li H, Durbin R. Fast and accurate short read alignment with Burrows-Wheeler transform. Bioinformatics. 2009; 25(14);1754–60. 10.1093/bioinformatics/btp324 19451168 PMC2705234

[32] McKenna A, Hanna M, Banks E, Sivachenko A, Cibulskis K, Kernytsky A, et al. The Genome Analysis Toolkit;a MapReduce framework for analysing next-generation DNA sequencing data. Genome Res. 2010; 20(9):1297–303. 10.1101/gr.107524.110 20644199 PMC2928508

[33] Robinson JT, Thorvaldsdóttir H, Winckler W, Guttman M, Lander ES, Getz G, et al. Integrative genomics viewer. Nature biotechnology. 2011 1 10;29(1):24. 10.1038/nbt.1754 21221095 PMC3346182

[34] Berger W, Meindl A, Van de Pol TJ, Cremers FP, Ropers HH, Döerner C, et al. Isolation of a candidate gene for Norrie disease by positional cloning. Nature genetics. 1992 6;1(3):199. 10.1038/ng0692-199 1303235

[35] Adzhubei I, Jordan DM, Sunyaev SR. Predicting functional effect of human missense mutations using PolyPhen-2. Curr Protoc Hum Genet. 2013;Chapter 7:Unit7 20. 10.1002/0471142905.hg0720s76 23315928 PMC4480630

[36] Zerbino DR, Achuthan P, Akanni W, Amode MR, Barrell D, Bhai J, et al. Ensembl 2018. Nucleic Acids Research. 2017 11 16;46(D1):D754–61. 10.1093/nar/gkx1098 PMC5753206 29155950

[37] Choi Y, Chan AP. PROVEAN web server: a tool to predict the functional effect of amino acid substitutions and indels. Bioinformatics. 2015 4 6;31(16):2745–7. 10.1093/bioinformatics/btv195 25851949 PMC4528627

[38] Yachdav G, Kloppmann E, Kajan L, Hecht M, Goldberg T, Hamp T, et al. PredictProtein—an open resource for online prediction of protein structural and functional features. Nucleic Acids Res. 2014 7;42(Web Server issue):W337–43. 10.1093/nar/gku366 Epub 2014 May 5. ; PMCID: PMC4086098. 24799431 PMC4086098

[39] Waterhouse Andrew, Bertoni Martino, Bienert Stefan, Studer Gabriel, Tauriello Gerardo, Gumienny Rafal, et al. SWISS-MODEL: homology modelling of protein structures and complexes, Nucleic Acids Research, Volume 46, Issue W1, 2 7 2018, Pages W296–W303 10.1093/nar/gky427 29788355 PMC6030848

[40] Amberger J, Bocchini CA, Scott AF, Hamosh A. McKusick’s online Mendelian inheritance in man (OMIM®). Nucleic Acids Research. 2008 10 7;37(suppl_1):D793–6. 18842627 10.1093/nar/gkn665 PMC2686440

[41] Stelzer G, Plaschkes I, Oz-Levi D, Alkelai A, Olender T, Zimmerman S, et al. VarElect: the phenotype-based variation prioritizer of the GeneCards Suite. BMC Genomics. 2016 6;17(2):444. 10.1186/s12864-016-2722-2 27357693 PMC4928145

[42] Daiger SP, Sullivan LS, Bowne SJ, Rossiter BJ. RetNet: Retinal Information Network. Na+ Ca2. 2013 6;5.

[43] Cunningham F, Amode MR, Barrell D, Beal K, Billis K, Brent S, et al. Ensembl 2015. Nucleic Acids Research. 2015 1 28;43(D1):D662–9. 10.1093/nar/gku1010 25352552 PMC4383879

[44] Yates A, Akanni W, Amode MR, Barrell D, Billis K, Carvalho-Silva D, et al. Ensembl 2016. Nucleic Acids Research. 2016 1 4;44(D1):D710–6. 10.1093/nar/gkv1157 26687719 PMC4702834

[45] Hitti RJ, Oliver JA, Schofield EC, Bauer A, Kaukonen M, Forman OP, et al. Whole genome sequencing of Giant Schnauzer Dogs with progressive retinal atrophy establishes NECAP1 as a novel candidate gene for retinal degeneration. Genes. 2019 5;10(5):385. 10.3390/genes10050385 31117272 PMC6562617

[46] Broeckx BJ. The dog 2.0: Lessons learned from the past. Theriogenology. 2020 1 21. 10.1016/j.theriogenology.2020.01.043 32000992

[47] Zhu M, Zhao S. Candidate gene identification approach: progress and challenges. International Journal of Biological Sciences. 2007;3(7):420. 10.7150/ijbs.3.420 17998950 PMC2043166

[48] Schaid DJ, Sommer SS. Genotype relative risks: methods for design and analysis of candidate-gene association studies. American Journal of Human Genetics. 1993 11;53(5):1114. 8213835 PMC1682319

[49] Tabor HK, Risch NJ, Myers RM. Candidate-gene approaches for studying complex genetic traits: practical considerations. Nature Reviews Genetics. 2002 5;3(5):391–7. 10.1038/nrg796 11988764

[50] Van Dijk EL, Auger H, Jaszczyszyn Y, Thermes C. Ten years of next-generation sequencing technology. Trends in Genetics. 2014 9 1;30(9):418–26. 10.1016/j.tig.2014.07.001 25108476

[51] Ohlmann A, Merkl R, Tamm ER. Focus on molecules: norrin. Experimental Eye Research. 2012 9 1;102:109–10. 10.1016/j.exer.2011.06.016 21736877

[52] Ke J, Harikumar KG, Erice C, Chen C, Gu X, Wang L, et al. Structure and function of Norrin in assembly and activation of a Frizzled 4–Lrp5/6 complex. Genes & Development. 2013 11 1;27(21):2305–19. 10.1101/gad.228544.113 24186977 PMC3828517

[53] Karczewski K.J., Francioli L.C., Tiao G. et al. The mutational constraint spectrum quantified from variation in 141,456 humans. Nature 581, 434–443 (2020). https://doi-org.ezp.lib.cam.ac.uk/10.1038/s41586-020-2308-7 32461654 10.1038/s41586-020-2308-7 PMC7334197

[54] Dickinson JL, Sale MM, Passmore A, FitzGerald LM, Wheatley CM, Burdon KP, et al. Mutations in the NDP gene: contribution to Norrie disease, familial exudative vitreoretinopathy and retinopathy of prematurity. Clinical & Experimental Ophthalmology. 2006 9;34(7):682–8. 10.1111/j.1442-9071.2006.01314.x 16970763

[55] Vallat M, Mathon C, Vancoppenolle F, Detre J, Fritsch D, Clavel D, et al. Retinopathy of prematurity-retinal-detachment in late stages. Journal Fransais D Ophthalmologie. 1986 1 1;9(8–9):573–82. 3819331

[56] Gilmour DF. Familial exudative vitreoretinopathy and related retinopathies. Eye. 2015 1;29(1):1. 10.1038/eye.2014.70 25323851 PMC4289842

[57] Shields JA, Shields CL, Honavar SG, Demirci H. Clinical variations and complications of Coats disease in 150 cases: the 2000 Sanford Gifford Memorial Lecture. American Journal of Ophthalmology. 2001 5 1;131(5):561–71. 10.1016/s0002-9394(00)00883-7 11336930

[58] Redmond RM, Vaughan JI, Jay M, Jay B. In-utero diagnosis of Norrie disease by ultrasonography. Ophthalmic Paediatrics and Genetics. 1993 1 1;14(1):1–3. 10.3109/13816819309087615 8345950

[59] Scott JP. Critical periods in the development of social behavior in puppies. Psychosomatic Medicine. 1958 1 1;20(1):42–54. 10.1097/00006842-195801000-00005 13505986

[60] Luhmann UF, Lin J, Lammel S, Feil S, Hammes HP, Berger W. Differential expression of angiogenic factors during postnatal retinal vascular development in Norrie disease mice. Investigative Ophthalmology & Visual Science. 2004 5 1;45(13):496-.

[61] Berger W, van de Pol D, Bächner D, Oerlemans F, Winkens H, Hameister H, et al. An animal model for Norrie disease (ND): gene targeting of the mouse ND gene. Human Molecular Genetics. 1996 1 1;5(1):51–9. 10.1093/hmg/5.1.51 8789439

[62] Pelcastre EL, Villanueva‐Mendoza C, Zenteno JC. Novel and recurrent NDP gene mutations in familial cases of Norrie disease and X‐linked exudative vitreoretinopathy. Clinical & Experimental Ophthalmology. 2010 5;38(4):367–74. 10.1111/j.1442-9071.2010.02245.x 20491809

[63] Meindl A, Lorenz B, Achatz H, Hellebrand H, Schmitz-Valckenberg P, Meitinger T. Missense mutations in the NDP gene in patients with a less severe course of Norrie disease. Human Molecular Genetics. 1995 3 1;4(3):489–90. 10.1093/hmg/4.3.489 7795608

