# Correction: Identification of a variant in *NDP* associated with X-linked retinal dysplasia in the English cocker spaniel dog

**DOI:** 10.1371/journal.pone.0272477

**Published:** 2022-07-28

**Authors:** Hannah Joyce, Louise M. Burmeister, Hattie Wright, Lorraine Fleming, James A. C. Oliver, Cathryn Mellersh

There is an error in affiliation 1 for authors Hannah Joyce, Lorraine Fleming, and James A. C. Oliver. The correct affiliation 1 is: Department of Ophthalmology, Dick White Referrals, part of Linnaeus Veterinary Limited, Six Mile Bottom, Cambridgeshire, United Kingdom.
