# Supplementary material for: Identification of a variant in NDP associated with X-linked retinal dysplasia in the English cocker spaniel dog
Source: PLoS One. 2021 May 4;16(5):e0251071. doi: 10.1371/journal.pone.0251071 (PMC8096109; doi:10.1371/journal.pone.0251071)
Supplement: S1 Appendix — (DOCX) [file pone.0251071.s001.docx]

**S1 Appendix: Reaction mixes and thermal cycling parameters:** Sanger Sequencing of candidate variants

Primers were designed to flank each variant by at least 100 nucleotides (*NDP*: forward 5’-ACAGGGATGGAAAACAAGTTC-3’ and reverse 5’-TCATCTCTTCTCTCCCCAGAT-3’; *COL9A3*: forward 5’-GCTTCCAGGTGTGTAAGGTTT-3’ and reverse 5’-CACAGGAGCAGCAGAAAGTTA-3’and *FJX1*: forward 5’-GAAGTTGGCCGTCAGGTAGTC-3’ and reverse 5’AAGGTGAGCAGCGCCC-3’). Each variant was amplified using HotStarPlus Taq Polymerase (Qiagen): Reactions were carried out in 12 µL volumes comprising 2 µL DNA at approximately 10 ng/µL, 0.2 mM dNTPs, 1x PCR buffer, 0.83 µM each of forward and reverse primers and 0.025 U/µL polymerase. Thermal cyclcing comprised denaturation at 95°C for 5 minutes; 30 cycles at 95°C for 30 seconds, 57°C for 30 second, 72° for 30 seconds, and followed by an elongation step at 72°C for 5 minutes. PCR products were purified using Ampure XP beads (Beckam Coulter Life Sciences) or a MultiScreen PCRµ96 Filter Plate (Merck Millipore, Hertfordshire, United Kingdom ) and used as templates for Sanger Sequencing. using Big Dye^TM^ Teminator v3.1 Cycle Sequencing Kit (ThermoFisher) on an ABI 3130xl Genetic Analyser (Applied Biosystems) according to the manufacturer’s instructions. Sequencing data was analysed using the Staden Package [1].

[1] Bonfield JK, Smith KF, Staden R. A new DNA sequence assembly program. Nucleic Acids Research. 1995 Jan 1;23(24):4992-9.
